# Electrolytic Surface Treatment for Improved Adhesion between Carbon Fibre and Polycarbonate

**DOI:** 10.3390/ma11112253

**Published:** 2018-11-12

**Authors:** Jan Henk Kamps, Luke C. Henderson, Christina Scheffler, Ruud van der Heijden, Frank Simon, Teena Bonizzi, Nikhil Verghese

**Affiliations:** 1 SABIC, Plasticslaan 1, 4612PX Bergen op Zoom, The Netherlands; ruud.vanderheijden@sabic.com; 2 Institute for Frontier Materials, Carbon Nexus, Deakin University, 75 Pigdons Rd, Waurn Ponds, VIC 3216, Australia; luke.henderson@deakin.edu.au; 3 Leibniz-Institut für Polymerforschung Dresden e.V. (IPF), Hohe Straße 6, 01069 Dresden, Germany; scheffler@ipfdd.de (C.S.); frsimon@ipfdd.de (F.S.); 4 SABIC Technology Center, 6160 AL Geleen, The Netherlands; Teena.Bonizzi@sabic.com; 5 SABIC Technology Center, Sugar Land, Houston, TX 77478, USA; nikhil.verghese@sabic.com

**Keywords:** carbon fibre, surface treatment, polycarbonate, composites, interfacial adhesion, single fibre pull out

## Abstract

To achieve good mechanical properties of carbon fibre-reinforced polycarbonate composites, the fibre-matrix adhesion must be dialled to an optimum level. The electrolytic surface treatment of carbon fibres during their production is one of the possible means of adapting the surface characteristics of the fibres. The production of a range of tailored fibres with varying surface treatments (adjusting the current, potential, and conductivity) was followed by contact angle, inverse gas chromatography and X-ray photoelectron spectroscopy measurements, which revealed a significant increase in polarity and hydroxyl, carboxyl, and nitrile groups on the fibre surface. Accordingly, an increase in the fibre-matrix interaction indicated by a higher interfacial shear strength was observed with the single fibre pull-out force-displacement curves. The statistical analysis identified the correlation between the process settings, fibre surface characteristics, and the performance of the fibres during single fibre pull-out testing.

## 1. Introduction

Improving mechanical properties through the addition of reinforcing fibres is a common approach used in a range of thermoplastic materials [1,2,3,4]. An important parameter faced in all research in this domain is the role of the interface between the fibre and the resin. To enable and exploit the mechanical property profile of fibre-reinforced thermoplastic composites, fibre-matrix adhesion must be at an optimum level [1,2,3]. An increase in the adhesion between carbon fibres and the polymer matrix can be achieved using different approaches, which are summarized in detailed review articles [5,6]. In general, wet-chemical (sizing/polymer finish, acidic modification, and electrochemical modification), dry-chemical (plasma/high energy irradiation modification, nickel surface coating, and thermal modification) and multiscale methods by applying nano-particles onto the surface are used to modify the carbon fibre surface. Each combination of fibre and matrix material will have its own ideal approach; for polycarbonate, specific studies have been conducted, mainly with respect to oxygen plasma-treated carbon fibres [7,8,9] or electrochemical oxidation [10,11,12,13], generally showing a significant increase in adhesion to polycarbonate after treatment. Table 1 gives an overview of the studies documented in literature and their results. The interfacial shear strength characterization can be approached using different micromechanical testing methods, each of which has its own unique procedure [14,15,16,17,18]. In this study, we focused on the single fibre pull-out, which is very suitable for evaluating the interfacial shear strength on a microscopic scale involving viscous polymers like polycarbonate.

Combining the modification of carbon fibres with surface characterization, followed by single fibre pull-out testing gives a detailed insight into the crucial parameters that control the interface and its impact on composite performance. Linking this approach with statistical studies to demonstrate the value of surface treatment for interface formation and compatibility of specific process settings with polycarbonate completes the work documented here.

## 2. Materials and Methods

The carbonization of poly (1-acrylonitrile) fibre, followed by varying surface treatments (adjusting the current, potential, and conductivity), created a range of seven different fibre samples used in this study (Table 2).

For the manufacture of carbon fibres in this study, three surface treatment variables were taken into account, which are thought to affect fibre-to-matrix adhesion. These include the current passed through the fibre during surface treatment, the potential applied to the fibre, and the conductivity of the electrolyte used in the bath (in this instance, ammonium hydrogen carbonate, [NH_4_]^+^ [HCO_3_]^−^).

It should be noted that due to the continuous nature of this production methodology, and the fact that the electrolyte is partially consumed during the surface treatment process, maintaining the exact level of each amperage, potential, and conductivity is challenging. Thus, different experiments used values as similar as possible for each of these, and these variables were classed into ‘bands’ of low, medium, and high for current (8, 14, and 26 A, respectively), and potential (5.7–5.8, 8–8.1, and 12.5–13.5 V, respectively). Only medium and high conductivities (17.0–17.5, and 31.2–31.4 mS/cm^−1^, respectively) were investigated, as low conductivity of the electrochemical bath carries a risk of equipment malfunction or breakage. 

To minimize the effect of unknown influences, a control sample (sample number 1), which did not have any surface treatment applied, was included in the experiment. This sample was collected directly after being passed through the high-temperature furnace.

LEXAN™ HF1110, a polycarbonate homopolymer (BPA) produced on a commercial scale by SABIC (Saudi Basic Industries Corporation, Riyadh, Saudi Arabia) and available as high-flow general-purpose grade, was used for the single fibre pull-out (SFPO) testing, selected for its lower viscosity, enabling efficient fibre embedding.

### 2.1. Inverse Gas Chromatography (IGC)—Surface Free Energy Analysis (SEA)

A series of *n*-alkanes (*n*-hexane, *n*-heptane, *n*-octane, and *n*-nonane) and polar probes (chloroform, ethyl acetate, 1,4-dioxane, ethanol, and dichloromethane) were injected into a column, which was filled with the fibre samples with specific fractional surface coverages, and their retention times were measured. The retention times (*t*) were converted into retention volumes. The dispersive free surface energy ($\gamma_{S}^{D}$) and specific free energy of desorption (${\Delta G}_{\mathrm{SP}}^{0}$) values on the surface of the fibre samples were determined in accordance with the standard method described by Jones [20].

The (${\Delta G}_{\mathrm{SP}}^{0}$) value obtained from the chloroform and ethyl acetate pair of mono-functional acidic and basic probes was used to determine the acid and base properties of the samples by applying an acid-base theory developed by van Oss [18,21]. The specific component of the surface free energy ($\gamma_{S}^{\mathrm{AB}}$) was calculated for this acid (Lewis electron pair acceptor) base (Lewis electron pair donor) pair. The so-called term ‘specific component of the surface free energy’ is widely used. However, according to the definition of the surface free energy {(∂f/∂o) = free energy (f), which is necessary to increase the surface (o)} it seems to be wrong to separate the surface free energy into dispersive and specific components because the surface free energy is an intrinsic value of the solid surface and does not depend on interacting liquids. The energy, which is determined from a solid surface coming into contact with a liquid, must be considered as interaction energy (both two phases contribute to the interaction energy). However, there is no problem in determining the interaction energy using IGC and splitting the interaction energy values into the contributions of dispersive (interaction) energy and specific (interaction) energy values.

The total surface free energy, $\gamma_{S}^{T}$, was calculated as the sum of the dispersive ($\gamma_{S}^{D}$) and specific ($\gamma_{S}^{\mathrm{AB}}$) energy contributions. Fitting the data to an exponential decay function, y = y_0_ + A exp [−x·t^−1^], allowed for extrapolation across the entire range (0–100%) of the surface coverage (x), where y_0_ is the value of the function at infinity.

### 2.2. Tensile Testing

Bare fibre samples were tested using a Favimat+ Robot 2 single fibre tester (Textechno H. Stein, Mönchengladbach, Germany) which automatically records linear density and force extension data for individual fibres loaded into a magazine (25 samples) with a pretension weight of 80 ± 5 mg attached to the bottom of each carbon fibre. Linear density was recorded using a length of 25 mm and a tension of 0.15 mN (as per the supplier specifications). The tensile load extension curves were collected at 1.0 mm/min using a gauge length of 25 mm and a pretension of 1.0 cN/tex. The load data were normalized by dividing by the linear density to give the specific stress strain curves from which the tensile strength (ultimate specific stress or tenacity) and specific modulus could be determined.

### 2.3. Tensiometer: Contact Angle and Surface Free Energy

The contact angles (CA) of fibre samples 1–7 with deionized water (milli-Q) and 1-bromonaphthalene (97%, Sigma-Aldrich, Taufkirchen, Germany) were measured on a force tensiometer K100SF with LabDesk 3.2.2 software from KRÜSS GmbH (Hamburg, Germany), which was placed on a TS-150 LP dynamic antivibration system supplied by TABLE STABLE (Mettmenstetten, Switzerland). The measurements were performed at ambient conditions.

In each test, a force-displacement curve was recorded while immersing a fibre into one of the test liquids at a length of 5 mm with a speed of 3 mm/min and a data acquisition step of 0.02 mm. For the detection of the fibre (a sudden change in force), a detection speed of 6 mm/min was used and the detection sensitivity was set at 2 × 10^−5^–7.5 × 10^−5^ g. By regression of the force-displacement curve and extrapolation to 0 mm immersion depth, the wetting force F was determined and the advancing contact angle (θ_a_) was calculated with the Wilhelmy Equation:

cosθ_a_ = F/(Lσ) (1)

where σ is the total surface tension of the liquid (water = 72.8 mN/m, 1-bromonaphthalene = 44.6 mN/m) and L is the perimeter of the fibre based on the average fibre diameter determined for each sample during tensile testing (see Section 2.2).

For each sample, 10 fibres were tested per test liquid, resulting in an average θ_a_ per test liquid. The average θ_a_ with water and 1-bromonaphthalene were used to calculate the surface free energy (SFE) values of all the fibre samples, using the Owens, Wendt, Rabel, and Kaelble (OWRK) method [22]. The total SFE of each sample equals the sum of a polar (σ^P^) and dispersive surface energy component (σ^D^). The surface polarity was determined by taking the ratio—reflected as a percentage—of the polar component to the overall SFE.

### 2.4. Single Fibre Pull-Out Test (SFPO)

The interfacial adhesion strength between the fibre and matrix was evaluated by means of a SFPO using purpose-built embedding equipment constructed at IPF Dresden (Germany) [15,23]. Samples were prepared by accurately embedding one end of the selected single fibre in the matrix (perpendicularly) with a pre-selected embedding length l_e_ (l_e_ = 150 μm). For polycarbonate, an embedding temperature of 300 °C was required and embedding was carried out at controlled atmosphere and temperature. After embedding, the temperature was held at 300 °C for about 30 s, before cooling down to ambient temperature. The pull-out test was carried out with a force accuracy of 1 mN, a displacement accuracy of 0.07 μm, and a loading rate of 0.01 μm/s at ambient conditions (using a self-made pull-out apparatus). The force-displacement curves and the maximum force, F_max_, required for pulling the fibre out of the matrix were measured. After testing, the fibre diameter, d_f_, was measured using optical microscopy; l_e_ was determined using the force-displacement curve and cross-checked using scanning electron microscope (SEM) Ultra (Carl Zeiss AG, Oberkochen, Germany). The adhesion bond strength between the fibre and the matrix was characterized by the values of the apparent interfacial shear strength (τ_app_ = F_max_/(π × d_f_ × l_e_)). Other interfacial parameters (such as local interfacial shear strength τ_d_ and interfacial frictional stress τ_f_) were not considered in this work for analysing the fibre-matrix adhesion. Most of the curves did not follow the characteristic shape of the pull-out curve as described in Reference [18], meaning that the determination of the characteristic points for modelling (debonding force F_d_, minimum force after debonding based on friction F_b_) were not clearly identifiable [24,25]. Instead, the debonding work (from l_e_ = 0 to l_e_ at F_max_) and pull-out work (from l_e_ at F_max_ to maximum l_e_ achieved at complete fibre pull-out) were used for comparison. Each fibre/matrix combination was evaluated in about 15–20 single tests. The filament surface before and after the pull-out test was evaluated using (SEM).

### 2.5. X-ray Photoelectron Spectroscopy (XPS)

All the XPS studies were carried out by means of an Axis Ultra photoelectron spectrometer (Kratos Analytical, Manchester, UK), equipped with a monochromatic Al Kα (1486.6 eV) X-ray source of 300 W at 15 kV. A hemispheric analyzer set to pass energy of 160 eV for wide-scan spectra and 20 eV for high-resolution spectra was used to determine the kinetic energy of the photoelectrons. The sample (carbon fibre tow) was mounted on a sample holder using adhesive tape so that the analyzed area was over an opening in the sample holder, enabling exposure to the X-ray source during measurement. Although the carbon fibres were electrically conductive, a low-energy electron source in combination with a magnetic immersion lens was employed to avoid electrostatic charging of the sample that can occur by fixing the fibres on the sample holder with the insolating adhesive tape. All the recorded peaks were shifted by the same value to set the C 1s component peak of the saturated hydrocarbons to 285.00 eV. The quantitative elemental compositions were determined from the peak areas using experimentally determined sensitivity factors and the spectrometer transmission function. Kratos spectra deconvolution software was applied to the high-resolution spectra and the spectrum background was subtracted according to Shirley. The free parameters of the component peaks were their binding energy (BE), height, full width at half maximum, and the Gaussian-Lorentzian ratio.

### 2.6. Statistical Evaluation

The testing results are reported as single values or mean ± standard deviation when multiple repeat evaluations of the fibre sample were conducted. Table 3 lists the average values, standard deviation, and sample size for (Favimat) tensile testing, and the single fibre pull-out measurements. In the results section the average values, standard deviation, and sample size for the contact angle measurements, which are used to calculate the energy and adhesion values listed in Table 3. Inverse gas chromatography was practiced on a bundle of fibres, resulting in responses based on the surfaces of numerous individual filaments; in all cases the line fit had a R^2^ > 0.997, showing a good representation of the reported results. XPS was carried out by irradiating an area of approximately 3 mm^2^ of the analyzed fibre bundles. From this irradiated area, the spectrometer collects nearly all the photoelectrons leaving the sample surface, measures their kinetic energy, and uses them to draw the corresponding spectrum, which reflects the average of the analyzed area, representing a large number of filaments.

To compare the physical properties of the six surface-treated samples to the control, the Dunnett’s Method test was used, the Pearson correlation coefficient (r) was calculated, and the *p*-value for statistical significance was derived.

To evaluate the relationship between the IFSS and the surface treatment parameters (current, potential, and conductivity) a linear regression model was used with both univariate and multivariable results reported as a parameter estimate (95% confidence interval) with a *p*-value. The fit of the model was assessed visually, and no concerns were noted.

All the analyses were performed on JMP© Pro 13, SAS Institute Inc., Cary, NC, USA, and a *p*-value of less than 0.05 was considered as statistically significant.

## 3. Results

The work documented here spreads across different disciplines and techniques. An overview of the results of the production, fibre characterization, and single fibre pull-out testing are reported in Table 3.

### 3.1. Fibre Surface Treatment Results and Differences Observed

The characterization of the untreated fibres (sample 1) showed a tensile strength and Young’s modulus of 3.84 and 259.85 GPa, respectively. For a comparison with a commercial product, these properties are slightly superior compared to automotive grade carbon fibres (T300, tensile strength and Young’s modulus of 3.53 and 230 GPa, respectively). Samples 3 and 7 had a statistically significant increase in Young’s modulus, though elongation at break and tensile strength were unchanged. Further improvements were observed when both the potential and current through the fibre were increased, at the same conductivity, though again, the only statistically significant change compared to sample 1 was with respect to the Young’s modulus. Interestingly, further increasing the amperage and potential caused the Young’s modulus to decrease slightly (Table 3, sample 4), and increasing the conductivity (Table 3, sample 5) corresponded to no meaningful property changes, suggesting that there is an optimal ratio and interplay between these three variables and that more of each, or even one, does not correspond to improved properties. Reverting to medium amperage and potential, which showed promise in sample 3, but increasing conductivity (Table 3, sample 6), had the most beneficial effects on the performance characteristics. All three measured parameters showed statistically significant changes relative to sample 1. Finally, combining low amperage and potential with high conductivity showed excellent improvements in all properties, suggesting that conductivity assists in the influence of the electrochemical treatments.

The application of current appeared to influence the modulus, with lower current settings being associated with a higher modulus. However, this effect can be modified by the potential setting. In particular, when the potential setting is low, increasing the current is associated with a higher modulus, but when the potential setting is high, increasing the current is associated with a lower modulus.

**Figure 1:**
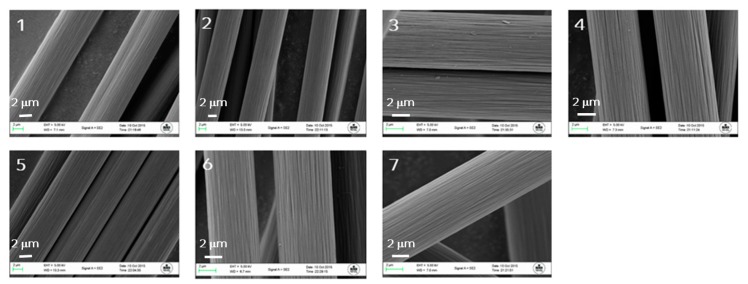
SEM images of all treated fibres from this study; sample 1 is the untreated sample, samples 2–7 show the same surface features and no surface defects have been detected.

The fibre surface was examined to ensure no pitting or surface defects had arisen on the fibre surface due to these oxidative procedures. Given the tensile strength data acquired for these samples, it is unlikely that any defects had been introduced to the surface; nevertheless, imaging the fibre using SEM was undertaken in the interest of thoroughness (Figure 1).

The visual examination of the fibres displayed no obvious changes compared to sample 1, which had not undergone any surface treatment. The longitudinal striations and fibre diameter (approx. 7 µm) were observed with all samples, suggesting that the surface treatment conditions, while aggressive in some instances, did not result in substantial fibre degradation. Given the consistency in surface structure and morphology, we turned our attention next to the examination of the surface chemistry using Inverse Gas Chromatography (IGC).

### 3.2. Treatment Impact on Surface Energy and Functional Groups, Matching with PC

**Figure 2:**
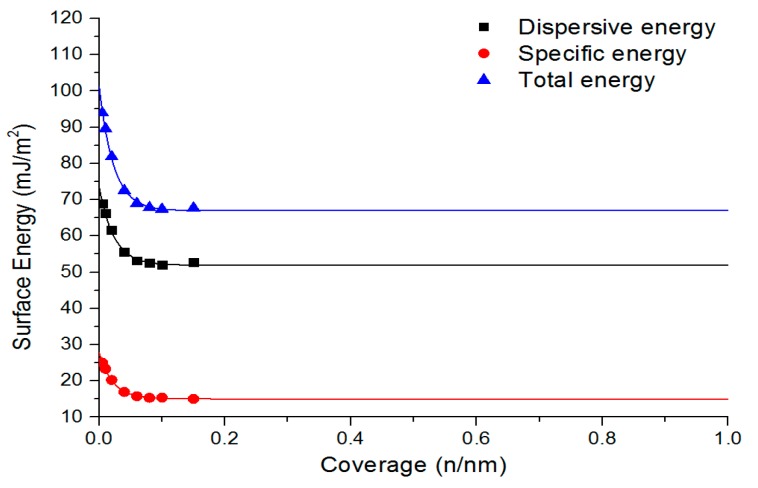
Inverse gas chromatography results (sample 1).

To determine the nature of the acid/base and the dispersive surface energies of the treated carbon fibres, we used IGC. In this technique, a column filled with the carbon fibres is injected with gaseous probes, which interact with various functional groups on the surface of the fibres. Typically, a range of non-polar (*n*-alkanes) and polar (ethyl acetate, ethanol, etc.) test liquids are used to determine the dispersive and Lewis acid/base properties, respectively (Table 4 and Figure 2).

Given the nature of this technique, a comparison of the absolute values is not informative, therefore the ratio of dispersive and polar energies is provided to give a more meaningful comparison between the samples. Sample 1, as expected, possessed a very high dispersive energy component, resulting from the highly graphitic nature of this fibre.

There is some evidence to suggest that increasing the current will decrease the dispersive surface energy and increase the specific surface energy, as can be observed in Table 4. Furthermore, the potential applied is likely to have a modifying effect on the surface properties. The similarity of the specific energy values for samples 3 and 4 is counter-intuitive considering that the oxidative treatment was more aggressive for sample 4 than for sample 3, suggesting that a plateau was reached under these conditions, perhaps dictated by the concentration, and thus conductivity, of the electrolyte.

A similar observation can be made when examining samples 5 and 6, where the polar portion of the surface energy remains at approximately 35–36% of the total surface energy, again suggesting a plateau of oxidative treatment and installation of polar functional groups. Interestingly, sample 7 shows a distinct decrease in polar surface energy (32%), relative to the other oxidized samples, which corresponds to a decrease in both current and applied potential. 

**Figure 3:**
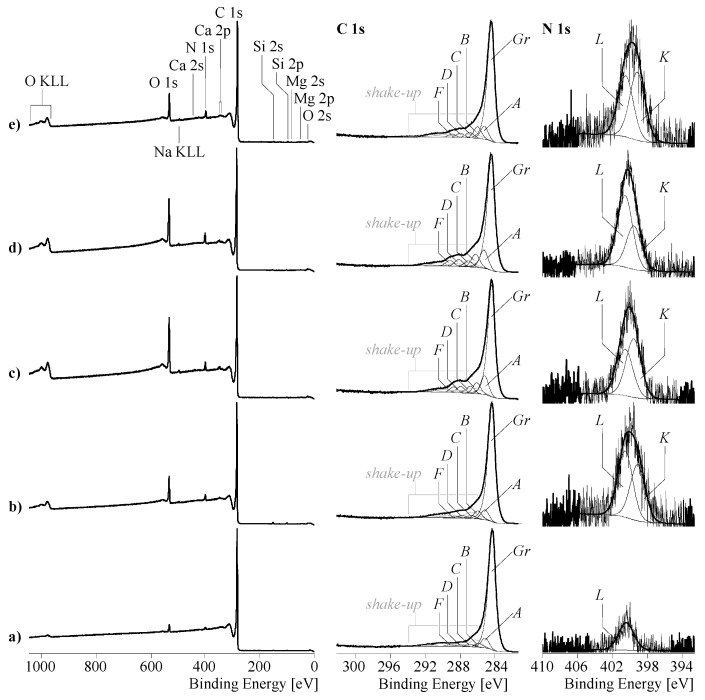
Wide-scan photoelectron spectra (left column), C 1s (middle column) and N 1s high-resolution photoelectron spectra (right column) recorded from unmodified carbon fibres (sample 1) (**a**), and electro-chemically modified carbon fibres at low current and low current and low conductivity (sample 2) (**b**), high current and low conductivity (sample 4) (**c**), high current and high conductivity (sample 5) (**d**), and low current and high conductivity (sample 7) (**e**).

While IGC thermodynamically described the interactions of solid surfaces to the probe molecules in their environment, XPS offered the opportunity to analyze the type and number of functional groups in the surface region of the differently treated carbon fibres. The wide-scan XPS spectra (Figure 3, left column) showed—with the exception of hydrogen—all the elements in the surface region of the carbon fibres. Besides the metal ions, such as sodium, magnesium, silicon, and calcium that occur only as traces (regarding carbon content, their contents were less than 0.5 at-%), considerable amounts of nitrogen and oxygen were detected on the carbon fibre surface.

Although oxygen may also be bonded in counter ions of the metal ions, it can be assumed that the majority of the oxygen atoms were covalently bonded to the carbon fibres. Nitrogen, which was found on the carbon fibre surfaces, could be a constituent of functional surface groups but also a residue of the ammonium salt (NH_4_^+^), which was used during the electrical oxidation process. Shape-analysis of the high-resolution element spectra is an established method to study the different binding states of the atoms in the surface region of solids. However, due to the so-called ‘shake-up’ phenomena, which were observed in XPS spectra recorded from substances consisting of graphite-like lattices, such as carbon fibres, the deconvolution of the C 1s spectra is generally difficult. Graphite-like lattices consist of sp^2^-hybridized carbon atoms in which the π-bonded p_z_-electrons can be extensively delocalized. Each linear combination of two of the p_z_ wave functions gives wave functions of one π-orbital occupied by the two p_z_-electrons and one unoccupied π*-orbital. In the case of graphite-like structures, the high number of possible linear combinations leads to a quasicontinuum of energy levels that can be occupied by electrons. Energy from an external source can be consumed to transfer a p_z_-electron from its π-orbital (ground state) into a π*-orbital (excited states). The C 1s spectra shows the photoelectrons emitted from the ground as well as excited states. The latter contribute to the shake-up peaks mentioned above.

The C 1s spectra recorded from all the carbon fibre samples are characterized by intense shake-up peaks appearing at binding energy values higher than 286 eV (Figure 3, middle column). In the same region, component peaks identifying different functional groups were expected. In order to separate the shake-up peaks overlapping the component peaks, it was assumed that the different surface modifications had the same effect on the π → π* transition probabilities and thus on the shape and intensities of all the shake-up peaks. The C 1s peak areas remained after subtraction, and the shake-up peaks were deconvoluted into six component peaks, showing different binding states of carbon. The most intense component peaks *Gr* (at 284.14 eV) resulted from the photoelectrons escaped from the sp^2^-hybridized carbon atoms, forming the graphite-like lattice of the carbon fibre material. Saturated hydrocarbons in the sp^3^ hybrid state, which did not have heteroatoms as binding partners, were assigned as component peaks *A* (at 285.00 eV). The presence of saturated hydrocarbons is frequently observed in surface analysis because non-specifically adsorbed contaminations mainly consist of alkanes and their derivatives. Component peaks *B* (at 285.84 eV) show C–N bonds of amines, C=N bonds of imines, and/or C≡N of nitrile groups. Surprisingly, the intensities of all component peaks *B* ([*B*]) were significantly higher than the [N]:[C] ratios independently determined from the wide-scan spectra ([*B*] ≈ 1.6 [N]:[C]). Obviously, considerable amounts of the nitrogen atoms were present as bound to two carbon atoms, which is well-known from the oxidized cyclization of the PAN structure before the carbonization process of the fibres [26]. The introduction of oxygen in the surface region of the carbon fibre samples resulted in the formation of C–O bonds of mainly phenolic groups (component peaks *C* at 286.69 eV), quinone-like groups (C=O as component peaks *D* at 287.77 eV), and carboxyl groups (O=*C*–OH) and their corresponding carboxylates (^−^O–C=O ↔ O=C–O^−^) both as component peaks *F* at 288.72 eV. Table 5 summarizes the fractions of the component peak areas and thus gives an overview of the number of different functional groups.

While the N 1s spectrum recorded from the unmodified carbon fibres showed a unimodal distribution of the photoelectrons around the peak maximum at 400.67 eV, the N 1s spectra of electro-chemically treated were deconvoluted into two component peaks, *K* and *L*. According to the binding energy values found for component peaks *L* (400.22 eV), it was assumed that these component peaks appeared from protonated nitrogen species, such as adsorbed ammonium ions (NH_4_^+^) or protonated amino groups (C–N^+^H). The component peaks *K* were found at about 399 eV, which is a very small value for organically bonded nitrogen. The chemical shift to small binding energy values indicated increased electron densities at the nitrogen atoms probably caused by C=N bonds of azoles [27] or azabenzenes in the immediate environment of highly conjugated π-electron systems, for example. In contrast, the binding energy for the triply bonded nitrogen in the nitrile groups (C≡N) is expected at 399.5 eV [28].

As *H*-acidic compounds, phenol and carboxyl groups are Brønsted and Lewis acids. Their deprotonated species, the phenolate and carboxylate ions, act as Brønsted and Lewis bases. Brønsted basic amino groups can be protonated by hydronium ions (H_3_O^+^). Amino, azole, and azabenzene groups belong to the group of nitrogen bases. Due to the −*I* effect of the nitrogen atom and the ability of nitrogen to bind a proton via its free electron pair, the nitrile group has an ambidentate character.

Contact angle measurements with a single fibre tensiometer resulted in a total surface free energy (SFE) and a surface polarity, which is the percentage of the total SFE that is due to the polar surface energy component, of the tested fibres (Table 6). All the surface-treated fibres had a numerically higher total SFE compared to the untreated fibre (sample 1, 41.9 mJ/m^2^), with fibre sample 4 having the highest value, 64.0 mJ/m^2^. In addition, all the surface-treated fibres had a higher surface polarity than the untreated fibre, and increasing the potential was associated with increasing polar surface energy.

**Figure 4:**
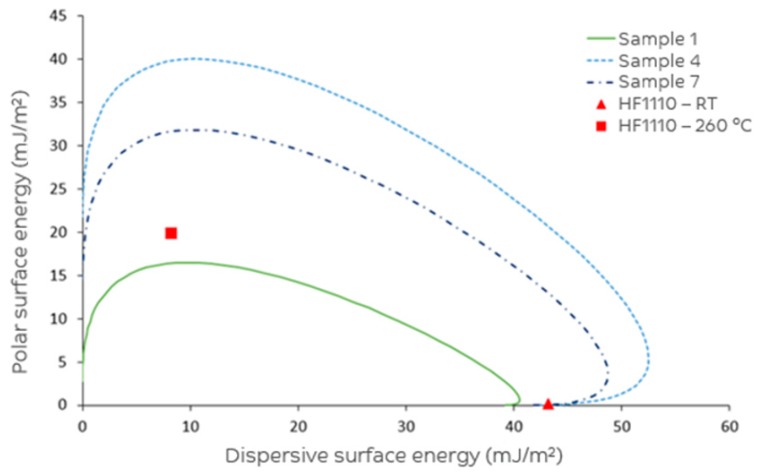
Wetting envelope for complete wetting (θ_a_ = 0°).

From the SFE values, a wetting envelope (Figure 4) for complete wetting could be calculated, which describes all the combinations of the polar (*y*-axis) and dispersive (*x*-axis) surface tensions of a liquid that would result in a θ_a_ of 0° by solving the OWRK equation. These wetting profiles allow for the prediction of the wetting behaviour of the fibres: the combinations inside the envelope will result in complete wetting (θ_a_ = 0°), while the combinations outside the envelope will not (θ_a_ > 0°). Figure 4 shows the wetting envelopes for the untreated fibre (sample 1) and the extremes of the treated fibres (samples 4 and 7). It can be seen that, theoretically, improved wetting can be expected of the surface-treated fibres with commercial LEXAN™ HF1110 polycarbonate at both ambient temperature (σ^P^ = 0.2 mJ/m^2^, σ^D^ = 43.2 mJ/m^2^) and 260 °C (σ^P^ = 19.9 mN/m, σ^D^ = 8.2 mN/m) compared to the untreated fibres.

To further quantify the compatibility with polycarbonate, the adhesion energy (ψ) and interfacial tension (γ) were calculated with the Fowkes/Dupre and Good’s expression, respectively, using the SFE values of the fibres and commercial LEXAN™ HF1110 [29]. The adhesion energy describes how energetically favourable the initial formation of an interface is, whereas the interfacial tension describes the tendency of the formed interface to break in the future upon stress. For good interfacial properties, high adhesion energy and low interfacial tension are targeted. Although it is assumed that the SFE values of the fibres are not dependent on the temperature, the total SFE and surface polarity of the polycarbonate matrix material changes when transitioning from a solid at ambient temperature to a molten polymer at 260 °C. Therefore, the interfacial parameters and trends amongst the fibre samples depend on the conditions used to combine the materials. Assuming a melt impregnation process, all surface-treated fibres show improved adhesion energy compared to the untreated fibre (50.6 mJ/m^2^), with the highest value being for fibre sample 4 (78.8 mJ/m^2^), which had the highest total SFE and surface polarity (Table 7). The conclusion as to how surface treatment influenced interfacial tension depends highly on the temperature studied: at ambient temperature, the untreated fibre looks superior, whereas at 260 °C all treated fibres are better than the untreated fibre. Single fibre pull-out testing has been attempted next to give more clarity as to which parameters and conditions are most indicative for optimal interfacial adhesion. 

### 3.3. Single Fibre Pull-Out Test (SFPO)

**Figure 5:**
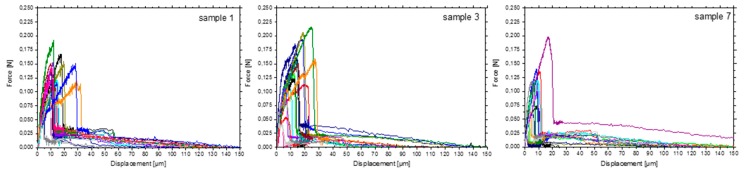
Force-displacement curves of sample 1 (no surface treatment), sample 3 (medium current, medium conductivity) as the most extensive, and sample 7 (low current, high conductivity) with the lowest fibre-matrix interaction, respectively.

Contact angle, IGC, and XPS measurements revealed a significant increase in the polarity and functional groups on the fibre surface due to the surface treatment. Accordingly, an increase in the fibre-matrix interaction indicated by higher maximum forces was observed using the SFPO force-displacement curves (Figure 5, showing three selected samples: untreated sample 1, highest (sample 3), and lowest (sample 7) maximum pull-out forces).

**Figure 6:**
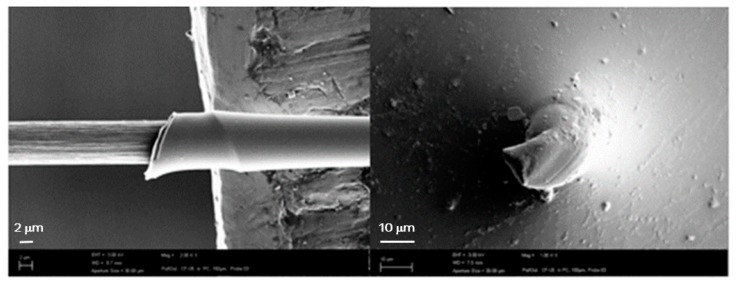
Stretching/yielding of the meniscus after pull-out testing; SEM image of the remaining part on the pulled-out fibre (**left**) and the stretched area of the fibre entry point in the PC droplet (**right**).

However, it should be noted that even the untreated fibre already reveals a good interaction between the fibre and the PC matrix. To some extent, this might be due to the fact that the PC matrix near the fibre is considerably deformed (stretched) during the pull-out. Figure 6 presents the deformation of the meniscus on the fibre surface as well as the strong deformation of the matrix material near to the fibre entry point. Besides the contribution of the crack that is growing along the fibre surface during pull-out and the friction between the already debonded surface areas, matrix deformation also contributes to the maximum force achieved. This would explain the high forces in the case of untreated sample 1.

On the contrary, this kind of meniscus stretching also occurred for sample 7, which revealed not only the lowest values of τ_app_ and W_debond_ but also a drop in polar surface; these might be related to each other. The currently known models (stress-controlled model; energy-based model, model of adhesion pressure [25]) used to calculate the interfacial parameters (ultimate interfacial shear strength τ_ult_, critical energy release rate G_ic_) do not involve this kind of meniscus deformation. As mentioned in Section 3.4, the apparent shear strength τ_app_ as well as debonding and pull-out work were used to describe the fibre-matrix interaction for that reason. In general, increased shear strength τ_app_ was found for the treated samples; however, the measurements were accompanied by high scatter due to the non-circular fibre shape.

### 3.4. Correlations

A statistical analysis was carried out to find the correlation between the process parameters, surface characterization techniques, and SFPO results and their significance (Table 8 and Table 9).

## 4. Discussion

Several approaches to modifying carbon fibre surfaces can be followed and their impact on adhesion to polycarbonate is studied, as listed in Table 1. In addition to the differences in testing methods, as described in the introduction, different suppliers and grades of polycarbonates were also used, where the difference in molecular weight will impact viscosity (and therefore wetting/impregnation) and the data comparison. This study focused on the electrolytic surface treatment of carbon fibre during its production process. Improvements in interfacial shear strength of comparable approaches have been documented [7,8,9,10,11,12,13], and fragmentation tests or indentation were used to quantify the impact. The authors of this article have also investigated the modification of polycarbonate as a means to achieving improved adhesion [19], where an apparent interfacial shear strength of 33.9 MPa was found for the reference polycarbonate (HF1110). By using functional groups, the adhesion was improved to 42.2 MPa, in combination with a commercially available carbon fibre (unsized), without further information on the specific process parameters of production.

By controlling the process parameters of the electrolytic surface treatment, a range of samples were created, which were characterized using chemical and mechanical characterization techniques, to evaluate the impact of treatment as well as the predictability of interfacial shear strength.

The mechanical properties of the fibres were in all cases equal or superior to untreated sample 1, showing a slight increase in Young’s modulus for samples 4 and 7, but no detrimental impact of the treatments were found and no obvious changes compared to sample 1 were observed from the SEM analysis.

From the inverse gas chromatography data, it would seem that the introduction of polar groups onto the surface of carbon fibres correlates well with the application of potential and current, which is in line with the observations made by contact angle measurements as well as the significant increase in the functional groups on the fibre surface observed using XPS.

There are challenges in controlling the exact level of amperage, potential, and conductivity applied to the samples, and when this is combined with the complexity of the analytical tests performed and a small sample size, establishing clear relationships between the process settings and the fibre characteristics was always going to be a challenge. However, we were able to demonstrate that current and potential are associated with a number of fibre features. In particular, we found that to influence τ_app_, there is an important interplay between the current and potential settings which means that tailoring these settings is not straightforward but that it could be possible to use this knowledge to target particular applications.

While significant correlations were found between the fibre characteristics, we did not find a direct correlation between process settings, tensile strength measurement, inverse gas chromatography or contact angle results and the single fibre pull-out parameters. This could be due to the limitations of the correlation test, assuming the relationships are linear. However, at this stage we were unable to find a test that correlates well with τ_app_, leaving the single fibre pull-out test as the most important analytical technique used in this study to predict interfacial shear strength.

The XPS characterization results did correlate significantly with the SFPO results. All the other techniques showed correlations among each other, but this did not render SFPO results (the most time-consuming and specialized technique) predictable enough for it to be acceptable to depend on it for research screening.

Further statistical evaluation of the presented dataset resulted in the predictive model for IFSS based on surface treatment process variables:

τ_app_ = (−0.32 × *V × I*) + (0.24 × *V × C*) + (2.1856 × *V*) + (2.4512 × *I*) − (2.4084 × *C*) + 48.7663 (2)

where *V* is the voltage applied, *I* is the value of current applied to the electrolytic solution, and *C* is the value of conductivity of the electrolytic solution. Using this formula to calculate the IFSS makes it possible to select the right process settings targeting a specific value.

**Figure 7:**
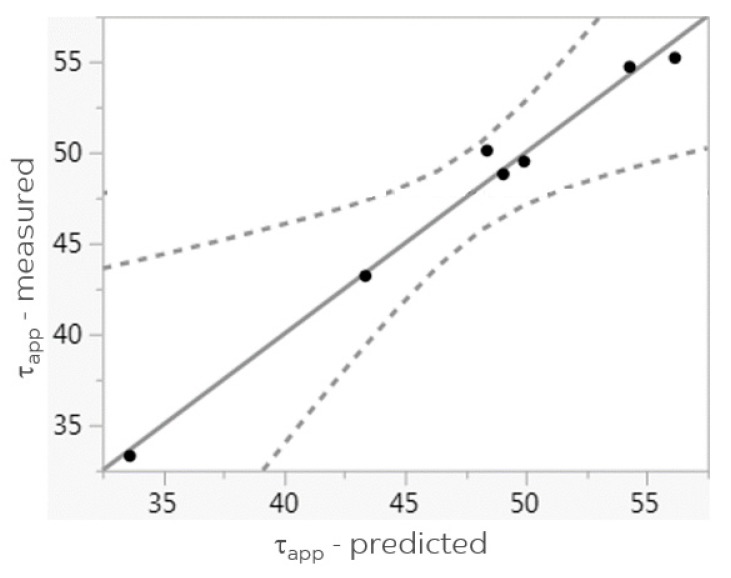
Linear correlation between τ_app_ predicted (Equation 2) and τ_app_ measured (samples 1–7).

Verification of this model, using the predicted values based on the process settings used in the preparation of samples 1 to 7 versus the actual measured values, shows a very good correlation (R^2^ = 0.99, *p* = 0.0255), as presented in Figure 7.

## 5. Conclusions

The impact of the electrolytic oxidation of carbon fibre on adhesion to polycarbonate has been studied and the impact of the variation of process parameters discussed. A set of on-purpose fibre samples were produced and characterized with a range of surface characterization techniques (IGC, XPS, CA), and single fibre pull-out testing was used for the quantification of the interfacial shear strength between the fibre and the polycarbonate matrix.

The statistical analysis showed significant correlations between IGC, XPS, and CA, but no predictive model was found in the pair-wise comparison between the surface characterization results and the single fibre pull-out measurements.

The dataset produced resulted in a predictive model for interfacial shear strength based on the process parameters used for electrolytic oxidation of the carbon fibre. This model makes it possible to target a certain interfacial shear strength, as desired or specified for the carbon fibre-polycarbonate composite.

## 6. Patents

The results of this study are documented in patent application “Methods for electrolytic surface treatment of carbon fibers”, USPTO serial number 62539879, published on IP.com with reference number IPCOM000252191D.

## Figures and Tables

**Table 1:** Overview of the material modifications, processing conditions, and micromechanical tests applied on carbon fibre—polycarbonate composites to increase and characterize the fibre-matrix adhesion by the interfacial shear strength (IFSS; the results represent the lowest and highest achieved value of the investigated materials for each reference); * *M*_w_ = molecular weight, ** SD = standard deviation.

Fibre	Treatment	Matrix	Testing Method	IFSS ± SD **	Ref.
PAN-based unmodified, unsized CF (Idemitsu Kosan, Tokyo, Japan)	Anodic oxidation (electrolyte solution: K_2_CO_3_/KOH; KNO_3_/KOH)	PC (Makrofol^®^, Bayer, Leverkusen, Germany)	Microdroplet pull-off test	9.6 ± 1.1 MPa (not oxidized); 14.7 ± 3.1 MPa (2.5 min in KNO_3_/KOH)	[10]
PAN-based CF with unknown sizing (12K, HTS40, Toho Inc. Corp., Tokyo, Japan) and self-prepared CF with epoxy sizing	Electrochemical oxidation using a 0.1 mol/L NaOH electrolyte	PC (Dongguang Plastic Film Corporation, Dongguang, China), focusing on polycarbonate backbone transesterification	Single fibre fragmentation test	25.04 ± 1.08 MPa (not oxidized); 47.53 ± 1.23 MPa (15 min treatment time)	[11]
PAN-based unmodified (UT) and oxidized (ST) CF (Toray Industries Inc., Tokyo, Japan)	Electrochemical oxidation	Bisphenol-A based PC with varying *M*_w_ * PC1 *M*_w_ 25,000 g/mol PC2 *M*_w_ 32,000–36,000 g/mol (consolidation temperature 230–310 °C)	Single fibre fragmentation test	PC 1: (230/310 °C) UT: 30.2/41.0 MPa ST: 43.8/56.5 MPa PC 2: UT: 42.8/48.4 MPa ST: 59.3/67.9 MPa	[12]
UHM pitch-based CF; HT PAN-based CF; both untreated and unsized	Microwave O_2_-plasma oxidation	PC Makrolon^®^ 2805 (Bayer, Leverkusen, Germany)	Single fibre fragmentation test	HT: 24.0 ± 2 MPa HT-ox.: 27.7 ± 2 MPa UHM: 12.2 ± 1 MPa UHM-ox: 46.7 ± 3 MPa	[7]
PAN-based CF (Hexcel Magnamite^®^ IM7, Stamford, CT, USA)	Commercial oxidative surface treatment at different grades	linear amorphous thermoplastic, Bisphenol-A based PC (GE Plastics, Inc., Pittsfield, MA, USA), *M*_w_ 31,000 g/mol	Microindentation test	100% ox.: 27.0 ± 1.9 MPa 400% ox.: 28.6 ± 3.2 MPa	[13]
PAN-based CF, Magnamite AS1 and AS4 (Hercules Aerospace, Wilmington, NC, USA)	Plasma treatment with ammonia, argon, nitrogen and oxygen	Polycarbonate LEXAN™ 101, (SABIC, Bergen op Zoom, The Netherlands)	Single fibre fragmentation test	lc/d 102% for ammonia treated lc/d 100% for ammonia treated lc/d 90% for argon treated lc/d 65% for oxygen treated	[8]
PAN-based CF, C320.00A, Sigri SGL Carbon, Wiesbaden, Germany	Low pressure oxygen plasma	PC Macrofol^®^ DE 1-1 (Bayer AG, Leverkusen, Gerrmany)	Single fibre fragmentation test	11.1 ± 1.2 MPa (no treatment) 9.8 ± 1.4 MPa (20 min treatment)	[9]
PAN-based CF, unsized	Commercial process, undisclosed	Functionalized polycarbonate (SABIC, Bergen op Zoom, The Netherlands)	Single fibre pull-out	33.9 ± 9.1 MPa (reference) 42.2 ± 9.0 MPa (functionalized PC)	[19]

**Table 2:** Surface treatment parameters and physical properties of resultant fibres.

Sample Number	Current (A)	Potential (V)	Conductivity (mS/cm)
1	-	-	-
2	8	5.8	17.5
3	14	8	17.5
4	26	13.5	17
5	26	12.5	31.3
6	14	8.1	31.4
7	8	5.7	31.2

**Table 3:** Complete overview of process settings and associated test results.

Sample Number	1	2	3	4	5	6	7	Testing Method
Current (A)	0	8	14	26	26	14	8	-
Potential (V)	0	5.8	8	13.5	12.5	8.1	5.7	-
Conductivity (mS/cm)	0	17.5	17.5	17	31.3	31.4	31.2	-
Elongation at Break (%)	1.58	1.60	1.63	1.64	1.70	1.79	1.63	Favimat
Standard deviation (n = 25)	0.24	0.29	0.24	0.28	0.32	0.23	0.24	-
Modulus (GPa)	259.85	261.44	266.24	262.06	261.81	263.09	264.22	Favimat
Standard deviation (n = 25)	3.59	4.58	11.36	3.26	5.20	4.73	4.26	-
Tensile strength (GPa)	3.84	3.88	4.05	4.02	4.13	4.38	4.00	Favimat
Standard deviation (n = 25)	0.61	0.72	0.62	0.71	0.80	0.58	0.62	-
Diameter (µm)	6.54	6.54	6.5	6.55	6.59	6.52	6.56	Favimat
Standard deviation (n = 25)	0.14	0.15	0.13	0.13	0.11	0.19	0.15	-
Total surface energy (mJ/m^2^)	67.0	68.1	72.2	75.7	73.2	72.7	70.5	IGC
Dispersive surface energy (mJ/m^2^)	51.9	47.4	46.1	47.8	46.9	46.4	47.4	IGC
Specific surface energy (mJ/m^2^)	15.0	20.6	26.0	27.5	26.0	26.1	22.7	IGC
Atomic Conc. Hydroxyl (%)	1.50	1.90	2.15	3.68	3.24	3.36	3.10	XPS
Atomic Conc. Carboxyl (%)	1.10	1.51	1.62	2.93	3.05	2.15	1.80	XPS
Atomic Conc. Nitrile (%)	2.07	4.79	4.48	5.75	7.17	6.52	6.70	XPS
Total surface energy (mJ/m^2^)	41.9	55.9	56.0	64.0	56.5	58.4	56.2	CA
Polar surface energy (mJ/m^2^)	2.7	14.8	17.2	21.8	20.3	18.2	14.4	CA
Dispersive surface energy (mJ/m^2^)	39.2	41.1	38.8	42.2	36.2	40.3	41.8	CA
Polarity (%)	6.5	26.4	30.7	34.0	35.9	31.1	25.6	CA
Adhesion energy ambient (mJ/m^2^)	83.7	87.6	85.5	89.5	83.1	87.2	88.3	CA
Interfacial tension ambient (mN/m)	1.6	11.6	13.9	17.9	16.8	14.7	11.3	CA
Adhesion energy 260 °C (mJ/m^2^)	50.6	71.0	72.6	78.8	74.6	74.4	70.9	CA
Interfacial tension 260 °C (mN/m)	19.4	13.0	11.4	13.3	9.9	12.2	13.4	CA
τ_app_ (N/mm^2^)	48.8	50.1	55.2	43.2	54.7	49.5	33.3	SFPO
Standard deviation (n = 25)	12.4	14.0	11.5	11.1	6.5	18.9	15.1	-
W_debond_ (mN mm)	1.5	1.2	1.8	0.7	0.9	0.7	0.6	SFPO
Standard deviation (n = 25)	0.9	0.6	1.3	0.9	0.6	0.5	0.6	-
W_pullout_ (mN mm)	2.1	1.6	1.5	2.9	2.2	1.3	2.0	SFPO
Standard deviation (n = 25)	0.9	0.6	0.7	2.7	2.8	0.7	1.1	-

**Table 4:** IGC results of the produced fibres.

Sample Number	Dispersive Energy (mJ/m^2^)	Specific (Acid-Base) (mJ/m^2^)	Total (mJ/m^2^)	Ratio of Dispersive and Specific Energies ^a^
1	51.94 (77.5%)	15.04 (22.5%)	66.98	3.45:1.0
2	47.41 (69.8%)	20.55 (30.2%)	68.14	2.31:1.0
3	46.06 (63.9%)	26.02 (36.1%)	72.19	1.77:1.0
4	47.83 (63.5%)	27.45 (36.5%)	75.72	1.74:1.0
5	46.88 (64.4%)	25.97 (35.6%)	73.23	1.81:1.0
6	46.40 (64.0%)	26.09 (36.0%)	72.65	1.78:1.0
7	47.38 (67.5%)	22.73 (32.5%)	70.49	2.08:1.0

^a^ Determined by dispersive/specific energies.

**Table 5:** Fractions of component peak areas.

Sample Number	[N]:[C]	[O]:[C]	[*B*]	[*C*]	[*D*]	[*F*]
1	0.011	0.022	0.021	0.015	0.008	0.011
2	0.030	0.084	0.048	0.019	0.017	0.015
3	0.028	0.105	0.045	0.022	0.021	0.016
4	0.036	0.163	0.058	0.037	0.034	0.029
5	0.045	0.142	0.072	0.032	0.038	0.031
6	0.042	0.107	0.065	0.034	0.032	0.022
7	0.042	0.087	0.067	0.031	0.025	0.018

**Table 6:** Tensiometer results of the fibres produced.

Sample Number	θ_a_ [Water] ^a^ (°)	θ_a_ 1-[Bromonaphthalene] ^a^ (°)	Total SFE (mJ/m^2^)	Surface Polarity (%)
1	82.6 ± 3.2 ^b^	28.7 ± 5.1	41.9	6.5
2	54.7 ± 3.9	22.2 ± 6.9	55.9	26.4
3	52.2 ± 3.8	29.1 ± 8.2	56.0	30.7
4	41.4 ± 3.7	17.8 ± 7.1	64.0	34.0
5	48.9 ± 4.5	35.8 ± 8.8	56.5	35.9
6	49.3 ± 4.4	24.4 ± 8.7	58.4	31.1
7	54.9 ± 4.5	19.7 ± 6.4	56.2	25.6

^a^ Based on 10 measurements. ^b^ Based on 8 measurements.

**Table 7:** Adhesion energy and interfacial tension for LEXAN™ HF1110 polycarbonate.

Sample Number	Adhesion Energy (mJ/m^2^)		Interfacial Tension (mN/m)	
	Ambient	260 °C	Ambient	260 °C
1	83.7	50.6	1.6	19.4
2	87.6	71.0	11.6	13.0
3	85.5	72.6	13.8	11.4
4	89.5	78.8	17.9	13.3
5	83.0	74.6	16.8	9.9
6	87.2	74.4	14.7	12.2
7	88.3	70.9	11.3	13.4

**Table 8:** Correlation factors on pair-wise comparisons between results, where 1 is the total positive linear correlation, 0 is no linear correlation, and −1 is the total negative linear correlation. The underlined factors show *p*-values < 0.05 and are considered as significant.

Factors	Elongation at Break	Modulus	Tensile Strength	Total Surface Energy	Dispersive Surface Energy	Atomic Conc. Hydroxyl	Atomic Conc. Carboxyl	Atomic Conc. Nitrile	Total Surface Energy	Polar Surface Energy	Dispersive Surface Energy	Polarity	Interfacial Tens. (Ambient)	Interfacial Tens. (260 °C)	τ_app_	W_debond_	W_pullout_
	Favimat			IGC		XPS			CA						SFPO		
Current (A)	0.46	0.17	0.61	0.92	−0.56	0.77	0.96	0.67	0.77	0.89	−0.21	0.86	−0.73	0.20	−0.40	0.45	0.46
Potential (V)	0.47	0.26	0.57	0.93	−0.66	0.80	0.93	0.73	0.87	0.95	−0.09	0.92	−0.79	0.13	−0.44	0.39	0.47
Conductivity (mS/cm)	0.72	0.45	0.16	0.51	−0.79	0.73	0.56	0.96	0.64	0.70	−0.06	0.75	−0.80	−0.18	−0.66	−0.21	0.72
Elongation at Break	-	0.20	−0.09	0.54	−0.56	0.66	0.52	0.67	0.45	0.55	−0.20	0.56	−0.55	0.18	−0.50	−0.32	1.00
Modulus	0.20	-	−0.39	0.38	−0.73	0.21	0.01	0.35	0.43	0.44	0.06	0.48	−0.56	−0.04	0.13	−0.39	0.20
Tensile strength	0.98	0.36	-	0.60	−0.63	0.67	0.50	0.66	0.50	0.59	−0.17	0.60	−0.59	0.17	−0.44	−0.34	0.98
Total surface energy (IGC)	0.54	0.38	0.60	-	−0.60	0.86	0.87	0.66	0.83	0.88	0.01	0.83	−0.65	0.01	−0.44	0.39	0.54
Dispersive surface energy (IGC)	−0.56	−0.73	−0.63	−0.60	-	−0.53	−0.45	−0.76	−0.79	−0.85	0.02	−0.90	0.94	−0.14	0.24	0.35	−0.56
Atomic conc. Hydroxyl (XPS)	0.66	0.21	0.67	0.86	−0.53	-	0.86	0.85	0.80	0.79	0.17	0.74	−0.57	−0.33	−0.83	0.37	0.66
Atomic conc. Carboxyl (XPS)	0.52	0.01	0.50	0.87	−0.45	0.86	-	0.74	0.72	0.82	−0.17	0.79	−0.64	0.04	−0.60	0.53	0.52
Atomic conc. Nitrile (XPS)	0.67	0.35	0.66	0.66	−0.76	0.85	0.74	-	0.76	0.81	−0.01	0.83	−0.81	−0.22	−0.76	0.04	0.67
Total surface energy (CA)	0.45	0.43	0.50	0.83	−0.79	0.80	0.72	0.76	-	0.95	0.34	0.91	−0.76	−0.15	−0.56	0.19	0.45
Polar surface energy (CA)	0.55	0.44	0.59	0.88	−0.85	0.79	0.82	0.81	0.95	-	0.03	0.99	−0.89	0.08	−0.46	0.15	0.55
Dispersive surface energy (CA)	−0.20	0.06	−0.17	0.01	0.02	0.17	−0.17	−0.01	0.34	0.03	-	−0.06	0.25	−0.73	−0.39	0.16	−0.20
Polarity (CA)	0.56	0.48	0.60	0.83	−0.90	0.74	0.79	0.83	0.91	0.99	−0.06	-	−0.95	0.14	−0.41	0.05	0.56
Interfacial tension (ambient) (CA)	0.55	0.42	0.59	0.89	−0.84	0.79	0.83	0.81	0.94	1.00	0.01	0.99	-	0.10	−0.46	0.16	0.55
Interfacial tension (260 °C) (CA)	−0.55	−0.56	−0.59	−0.65	0.94	−0.57	−0.64	−0.81	−0.76	−0.89	0.25	−0.95	−0.89	-	0.26	0.18	−0.55

**Table 9:** *p*-values for the correlation factors on pair-wise comparisons; *p*-values < 0.05 are considered as significant and are underlined.

Factors	Elongation at Break	Modulus	Tensile Strength	Total Surface Energy	Dispersive Surface Energy	Atomic Conc. Hydroxyl	Atomic Conc. Carboxyl	Atomic Conc. Nitrile	Total Surface Energy	Polar Surface Energy	Dispersive Surface Energy	Polarity	Interfacial Tens. (Ambient)	Interfacial Tens. (260°)	τ_app_	W_debond_	W_pullout_
	Favimat			IGC		XPS			CA						SFPO		
Current (A)	0.30	0.72	0.29	0.00	0.19	0.04	0.00	0.10	0.04	0.01	0.65	0.01	0.01	0.06	0.67	0.38	0.31
Potential (V)	0.29	0.58	0.27	0.00	0.10	0.03	0.00	0.06	0.01	0.00	0.85	0.00	0.00	0.04	0.79	0.32	0.39
Conductivity (mS/cm)	0.07	0.31	0.07	0.24	0.03	0.06	0.19	0.00	0.12	0.08	0.90	0.05	0.08	0.03	0.70	0.11	0.65
Elongation at Break	-	0.67	0.00	0.21	0.19	0.11	0.23	0.10	0.31	0.20	0.66	0.19	0.20	0.20	0.70	0.25	0.49
Modulus	0.67	-	0.42	0.41	0.06	0.65	0.99	0.44	0.33	0.32	0.90	0.27	0.34	0.19	0.93	0.79	0.39
Tensile strenght	0.00	0.42	-	0.15	0.13	0.10	0.26	0.11	0.26	0.17	0.71	0.15	0.17	0.17	0.72	0.33	0.45
Total surface energy (IGC)	0.21	0.41	0.15	-	0.16	0.01	0.01	0.11	0.02	0.01	0.98	0.02	0.01	0.11	0.98	0.32	0.39
Dispersive surface energy (IGC)	0.19	0.06	0.13	0.16	-	0.22	0.31	0.05	0.03	0.02	0.96	0.01	0.02	0.00	0.77	0.60	0.44
Atomic conc. Hydroxyl (XPS)	0.11	0.65	0.10	0.01	0.22	-	0.01	0.01	0.03	0.03	0.71	0.06	0.03	0.18	0.47	0.02	0.41
Atomic conc. Carboxyl (XPS)	0.23	0.99	0.26	0.01	0.31	0.01	-	0.06	0.07	0.02	0.71	0.04	0.02	0.12	0.93	0.15	0.22
Atomic conc. Nitrile (XPS)	0.10	0.44	0.11	0.11	0.05	0.01	0.06	-	0.05	0.03	0.99	0.02	0.03	0.03	0.63	0.05	0.93
Total surface energy (CA)	0.31	0.33	0.26	0.02	0.03	0.03	0.07	0.05	-	0.00	0.46	0.00	0.00	0.05	0.74	0.20	0.69
Polar surface energy (CA)	0.20	0.32	0.17	0.01	0.02	0.03	0.02	0.03	0.00	-	0.95	0.00	0.00	0.01	0.87	0.30	0.76
Dispersive surface energy (CA)	0.66	0.90	0.71	0.98	0.96	0.71	0.71	0.99	0.46	0.95	-	0.89	0.99	0.59	0.06	0.39	0.73
Polarity (CA)	0.19	0.27	0.15	0.02	0.01	0.06	0.04	0.02	0.00	0.00	0.89	-	0.00	0.00	0.77	0.36	0.91
Interfacial tension (ambient) (CA)	0.20	0.34	0.17	0.01	0.02	0.03	0.02	0.03	0.00	0.00	0.99	0.00	-	0.01	0.84	0.30	0.73
Interfacial tension (260 °C) (CA)	0.20	0.19	0.17	0.11	0.00	0.18	0.12	0.03	0.05	0.01	0.59	0.00	0.01	-	0.57	0.57	0.70
